# Natural Xylooligosaccharides Exert Antitumor Activity via Modulation of Cellular Antioxidant State and TLR4

**DOI:** 10.3390/ijms231810430

**Published:** 2022-09-09

**Authors:** Tsvetelina Batsalova, Yordan Georgiev, Dzhemal Moten, Ivanka Teneva, Balik Dzhambazov

**Affiliations:** 1 Faculty of Biology, Paisii Hilendarski University of Plovdiv, 4000 Plovdiv, Bulgaria; 2 Laboratory of Biologically Active Substances, Institute of Organic Chemistry with Centre of Phytochemistry, Bulgarian Academy of Sciences (IOCCP-BAS), 4000 Plovdiv, Bulgaria

**Keywords:** xylooligosaccharides, chemical characterization, antioxidant activity, in vitro cytotoxicity, antitumor activity

## Abstract

It has been recently proven that xylooligosaccharides (XOS) with prebiotic properties have diverse beneficial biological effects including immunomodulatory and antitumor activities. The present article focused on the chemical and biological evaluation of corn-derived commercially available XOS and aimed to elucidate their cytotoxicity and inhibitory potential against tumor cells. Spectrophotometric chemical analyses, Fourier transform infrared spectroscopy, and high-performance liquid chromatography analyses were performed. Antioxidant activity was determined by measuring the oxygen radical absorbance capacity and hydroxyl radical averting capacity. In vitro cytotoxicity assays with human cell lines derived from normal and tumor tissues, assessments of ATP production, mitochondrial membrane potential specific staining, cytokine assays, and molecular docking were used to evaluate the biological activity of XOS. The sample showed significant antioxidant activity, and it was determined that most xylose oligomers in it are composed of six units. XOS exhibited antitumor activity with pronounced inhibitory effect on lysosomes, but mitochondrial functionality was also affected. The production of proinflammatory cytokines by lipopolysaccharide-stimulated U-937 cells was reduced by XOS treatment, which suggested the involvement of Toll-like receptor 4 (TLR4)-mediated signaling in the mechanism of XOS action. Molecular docking analyses confirmed the potential inhibitory interaction between the sample and TLR4. In addition, XOS treatment had significant tumor-cell-specific influence on the glutathione antioxidant system, affecting its balance and thus contributing to the inhibition of cellular viability. The present study elucidated the tumor-inhibitory potential of commercially available XOS that could be utilized in pharmaceutical and food industry providing disease-preventive and therapeutic benefits.

## 1. Introduction

Xylooligosaccharides (XOS) are natural products with a degree of polymerization (DP) generally ranging from 2 to 10 or a maximum of up to 20 units [1]. They are produced by the breakdown of heteroxylan, which is the most abundant hemicellulosic fraction in plants playing different roles in the cell wall integrity, strength, protection, plant growth, and development [2]. Heteroxylans are composed of D-xylose residues linked together by *β*-(1→4)-glycosidic linkages, and they can be *O*-2/*O*-3-substituted with not only α-L-arabinofuranosyl residues but also *α*-d-glucuronic acid and/or its 4-*O*-methyl derivative at the *O*-2 position [3,4]. Methyl ethers and acetyl esters are found on xylose units, and arabinose residues can be decorated with ferulic and *p*-coumaric acids at the *O*-5 position [5].

XOS are produced from agro-industrial wastes, such as corn cob, wheat and rice straws, tobacco stalks, sugarcane bagasse, and brewers’ spent grains using chemical, physical, or enzymatic methods and their combinations [6,7,8,9,10,11]. XOS production by enzymatic hydrolysis with free or immobilized *endo*-1,4-*β*-d-xylanase, acting in a synergism with heteroxylan debranching enzymes, is a fast and more eco-friendly method compared with chemical and physical hydrolyses [5,12]. Enzyme synthesis of XOS with specific DP is also studied [13]. The enzyme modification does not accumulate unwanted byproducts; the yields of XOS are reasonable, but the main problems are enzyme cost, removal, stability, and reuse, as well as enzyme substrate specificities [5]. Researchers are focused on combining methods and testing different raw materials, extraction approaches, and enzymes to achieve a cost-effective and healthful product with optimal production efficiency.

XOS commercial production has begun since the 1980s in Japan, and the global XOS market size was estimated to be USD 99 million in 2019 and is expected to reach USD 128 million in 2025, with a compound annual growth rate of 4.4% (360 Research Reports, 2021). XOS are accepted as a novel food pursuant by the European Food Safety Authority (EFSA), and they are generally recognized as safe for use in foods by the Food and Drug Administration (FDA) in the USA exhibiting no toxic effects at a single dose of 5 g/kg body weight in Wistar rats [14]. They find application in functional and dietary nutrition because of their beneficial effects and excellent physicochemical properties, such as water solubility, low viscosity, pH stability (2.3–8.0), and thermostability [15]. XOS are classified as emerging prebiotics, which are not absorbed and digested by intrinsic enzymes in the intestine, but serve as a nutrient for commensal intestinal bacteria [16,17]. The oral administration of XOS in healthy individuals has increased *Bifidobacterium* counts without gastrointestinal side effects [18]. In a dose of 2 g/day for 8 weeks, XOS improved insulin sensitivity and reduced prediabetic gut microbiota in prediabetic patients [19]. The administration of 4 g/day XOS for 8 weeks has decreased blood glucose, HbA1c, and fructosamine concentrations, as well as total cholesterol, oxidized low-density lipoprotein (LDL), and apolipoprotein B in diabetic patients [20]. XOS have improved triglyceride, total cholesterol, LDL levels, and regulated mRNA expression of carnitine palmitoyltransferase 1, peroxisome proliferator-activated receptor-α, cytochrome P450 family 7 subfamily A1 (CPT-1, PPAR-α, and CYP7A1), acetyl-CoA carboxylase, and lipoprotein lipase in mice fed with a high-fat diet [21].

XOS have also manifested immunomodulatory effects by inhibiting the expression of proinflammatory mediators, such as tumor necrosis factor-α (TNF-α), interleukin-1β (IL-1β), IL-6, and NO, and induced IL-10 production in lipopolysaccharide-stimulated RAW264.7 macrophages [22]. They have attenuated systemic and colon inflammation in mice and obese rats and positively affected liver steatosis in rats, suppressing adipose tissue inflammation and improving insulin signaling [23,24,25].

Despite the accumulated scientific information so far, the biological activities of XOS are still not well-studied and are a challenge for many researchers. For example, several publications have demonstrated the role of XOS as cytotoxic agents against human leukemia cell lines, MCF-7 breast cancer cells, HT-29, and Caco-2 colorectal carcinomas in vitro [26,27,28]. An intake of 60 g XOS/kg diet has considerably reduced the formation of precancerous colon lesions in male Sprague–Dawley rats treated with the carcinogen 1,2-dimethylhydrazine [29]. Interestingly, the colon cancer prevention effects of XOS have been attributed to reduced lipid peroxidation and activation of glutathione-S-transferase and catalase in the colon mucosa and liver in rats [30]. This reveals the possible antioxidant defensive effects of XOS in carcinogenesis prevention. In fact, XOS have exhibited strong dose-dependent in vitro antioxidant effects by the 2,2-diphenyl-1-picrylhydrazyl (DPPH), 2,2′-azino-bis(3-ethylbenzothiazoline-6-sulfonic acid) (ABTS), ferric reducing antioxidant power (FRAP), and total antioxidant capacity (TAC) methods [10,14,31]. However, it has been found in many studies that XOS contain residual phenolic compounds, which needs the elucidation of the antioxidant potential of XOS alone. Despite the advances in research into the antitumor activity of XOS, the mechanisms and signaling pathways leading to these effects are still unclear. Further studies on the antitumor activity of XOS are needed. That is why the aim of the current study was to chemically characterize commercially available XOS and evaluate their antioxidant potential and cytotoxic effect against different human tumor cell lines in vitro.

## 2. Results

### 2.1. Chemical Characterization of the XOS Sample

The results from the chemical characterization of the XOS sample are summarized in Table 1. Its total carbohydrate content was 97.3% (*w/w*) with a negligible amount of uronic acids (<1%). Two monosaccharides were detected in the sample, and xylose (Xyl) was the predominant one (59.4%), as it was expected. The conditions of the acidic hydrolysis of poly- and oligosaccharides are extremely important for an adequate determination of their monosaccharide content. A mild hydrolysis with 0.2 M trifluoroacetic acid (TFA) and a stronger hydrolysis with 2 M TFA were conducted, but no difference in the xylose content was observed, which limited the possibility for considerable xylose degradation during the acidic treatments. Furthermore, the XOS sample also had 9.7% glucose, which was 10.6 ± 0.7% and 8.9 ± 0.4% after treatment with 2 and 0.2 M TFA, respectively. The presence of glucose could be explained by the source for XOS production (plant material) and the technology used for the preparation of oligosaccharides or the addition of food-grade fillers, such as dextrins (E1400) or starch. The qualitative test with Lugol′s iodine did not give a characteristic blue color, but the sample was colored in dark brown. Therefore, the presence of starch was excluded, but it was suggested that the sample contained glucose derived from dextrins. Interestingly, further analyses determined that the XOS sample had 0.6% acetyl content, which suggested that Xyl residues were acetylated. It was calculated that the degree of acetylation of Xyl units was 3.3 mol% on the basis of the Xyl content in the sample. Additionally, it was proven that the XOS sample did not contain proteins, which was important to be checked because of further biological studies.

**Figure 1:**
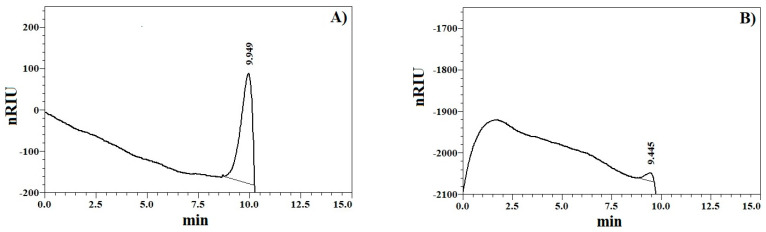
Elution profile of xylooligosaccharide sample (**A**) and 1,3:1,6-*β*-d-glucohexaose (**B**).

When comparing the high-performance size-exclusion chromatography-refractive index detection (HPSEC-RID) elution profile of XOS (Figure 1A) with those of pullulan standards, it was found that the sample was characterized with a molecular weight distribution lower than that of the smallest standard of 0.59 × 10^4^ g/mol. However, oligosaccharide standards, such as laminaritriose, laminaritetraose, laminaripentaose, and 1,3:1,6-*β*-D-glucohexaose, were also analyzed, and only the last oligosaccharide gave a distinctive peak with a retention time of 9.445 min (Figure 1B). Therefore, carbohydrates with a DP lower than 6 were not practically detectable. Interestingly, the XOS mixture was eluted at 9.949 min (Figure 1A); thus, it was suggested that the sample contained oligomers with a DP around 6 or more. This was in good agreement with the information from the certificate of the manufacturer stating a DP of XOS between 2 and 7. Additionally, no characteristic peak for a polymeric fraction, such as starch, was detected before the peak of XOS, which showed that the glucose in the sample most probably did not originate from starch. This confirmed the result from the performed qualitative test for starch.

Although the total phenolic content of the sample was negligible, it expressed detectable in vitro antioxidant activity through the oxygen radical absorbance capacity (ORAC) method and lower via the hydroxyl radical averting capacity (HORAC) method (Table 1). This revealed that XOS were able to scavenge the peroxyl and hydroxyl radicals generated in the in vitro environment. Phenolics in the XOS sample can derive from the residual lignin.

**Figure 2:**
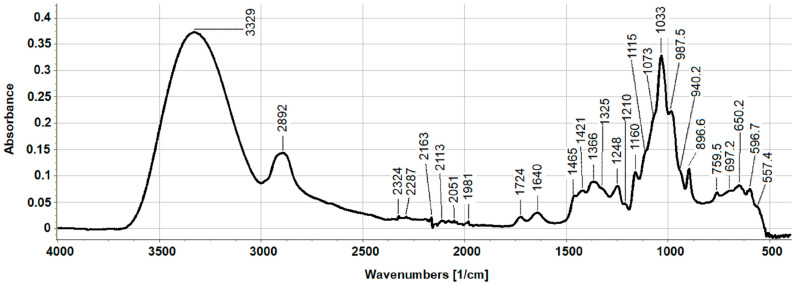
Fourier transform infrared spectrum of xylooligosaccharide sample obtained using attenuated total reflection technique.

Figure 2 shows the attenuated total reflectance–Fourier transform infrared spectroscopy (ATR-FTIR) spectrum of XOS. The spectral characteristic of the sample confirmed that it was of a carbohydrate nature because of the presence of typical absorption bands for sugars. The signals at 3329 and 2892 cm^−1^ were attributed to the specific vibrations of ν(OH) and $v{\left(\mathrm{CH}\right)}_{{\mathrm{CH}}_{2}}$, respectively, in the xylose and glucose rings [32].

The absorption bands at 1724 and 1248 cm^−1^ were linked to ν(C=O)_Ac_ and ν(C-O-C)_Ac_ stretching vibrations, respectively, confirming the existence of acetyl esters on Xyl residues, and the signal at 1640 cm^−1^ originated from water [32]. In the range from 1500 to 1600 cm^−1^, vibrations of the CO of COOH, NH_2_, and those of aromatic rings were not found. Particularly, the lack of signal at around 1500–1550 cm^−1^ for amide II structure (*δ*(NH) and *ν*(CN)) proved that the sample did not contain proteins. The absence of a characteristic band for the benzene nucleus also indicated that the XOS sample did not contain representative amounts of phenols. This was in agreement with the colorimetric determination of proteins and total phenolics in the sample. It was important to have, as much as possible, a low content of noncarbohydrate constituents in the sample because of the further studying of its biological activity. The bands at 1465, 1421, 1325, and 1248 cm^−1^ were associated with the presence of C–H and CO vibrations derived from hemicellulose XOS [33]. The peak at 1366 cm^−1^ is mainly assigned as the vibrations of the CH_3_ group, but in this case, it was a result of the oscillation of the C–H bond in glucose units in the sample [34]. In general, different types of carbohydrates differ in the characteristic or the so-called fingerprint region between 1000 and 1200 cm^−1^. The oscillations in the carbohydrate rings CO, CC, C–OH, and COC of the ether glycosidic bonds between the individual monosaccharides are found in this area. The combination of shoulder signals at 1160, 1115, and 988 cm^−1^ confirmed the predominance of Xyl units in the sample, which was consistent with the monosaccharide composition data [35]. The absorption band at 897 cm^−1^, originating from C_1_–OH stretching vibrations, showed the predominance of monosaccharides in *β*-anomeric configurations, which were Xyl residues.

### 2.2. XOS Cytotoxicity and Antitumor Activity In Vitro

**Figure 3:**
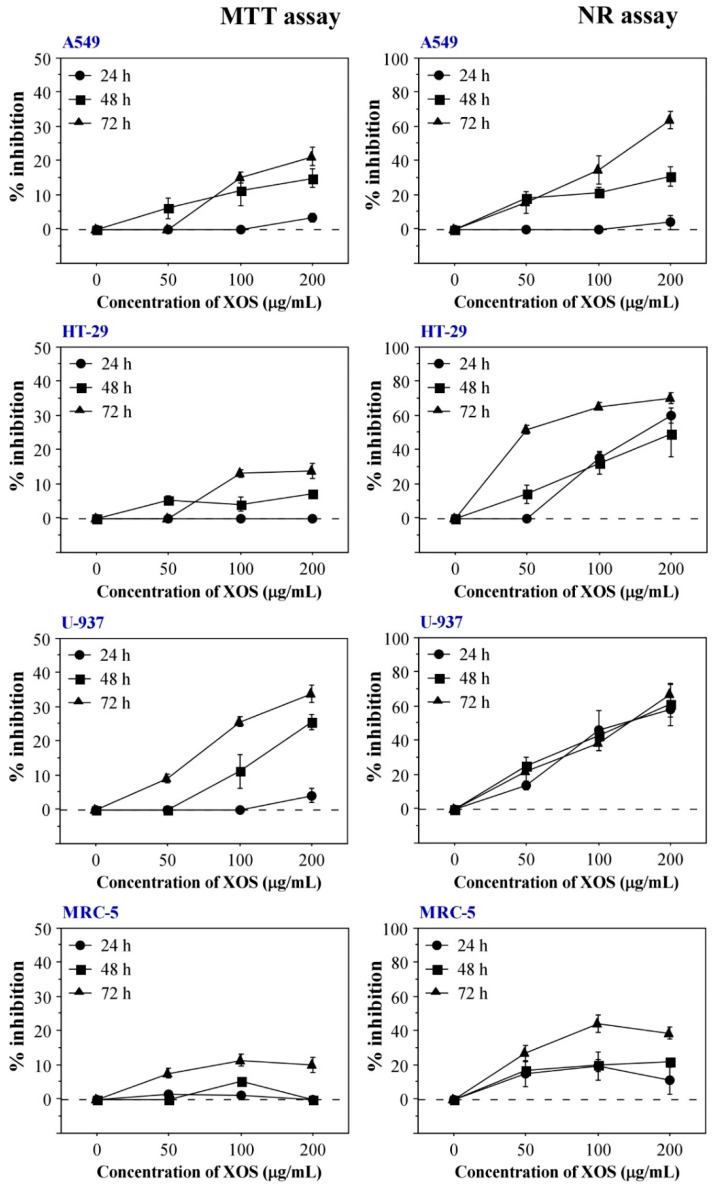
In vitro cytotoxicity of XOS determined by MTT and NR assays. Left column with graphs shows MTT assay results for XOS-treated cells from A549, HT-29, MRC-5, and U-937 cell lines. Graphs on the right represent data obtained from NR uptake tests with the same cell types. Results are presented as mean ± SEM. All samples were assayed in triplicates.

To determine if the XOS sample induces cytotoxic and/or antitumor effects, in vitro assays with human cell lines derived from normal tissue (MRC-5 lung fibroblasts) and different tumors (A549 lung adenocarcinoma, HT-29 colon adenocarcinoma, and U-937 histiocytic lymphoma) were performed. The evaluations were implemented via the 3-(4,5-dimethylthiazol-2-yl)-2,4-diphenyltetrazolium salt (MTT) assay and the neutral red (NR) uptake test. The main reason for the choice of these methods relates to their good sensitivity compared with other classical in vitro cytotoxicity assays [36]. Two different routes of cellular functionality following treatment with XOS were assessed—cellular viability and metabolic activity evaluated by the extent of MTT reduction, and lysosomal functionality based on the accumulation of NR stain. The obtained results are presented in Figure 3. They indicate an antitumor potential of the sample because XOS treatment led to stronger cytotoxic effects in tumor cell lines compared with the MRC-5 fibroblasts. Data from both in vitro assays showed that XOS induce time- and concentration-dependent cellular inhibition. Interestingly, NR assays showed higher levels of cellular inhibition compared with the MTT assay results suggesting a lysosome-specific mechanism of cytotoxicity. Half-maximal inhibitory concentrations (IC50) and selectivity indices (SI) were calculated based on the data from NR assays with different cell lines treated for 72 h with XOS (Table 2). All tumor cell types included in the experiments showed a SI higher than 2, which confirmed the tumor-specific cytotoxic effect of the XOS sample.

**Figure 4:**
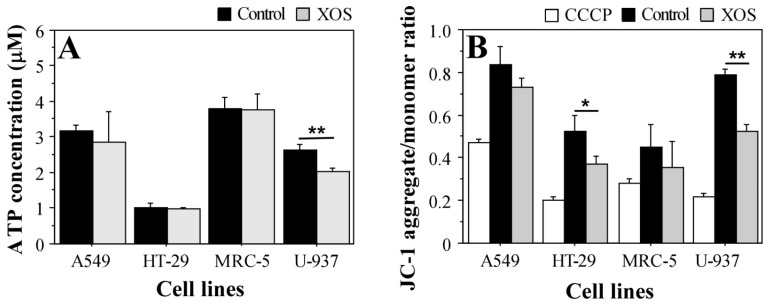
Intracellular ATP concentration (**A**) and mitochondrial transmembrane potential state (**B**) following 24 h treatment with 200 μg/mL xylooligosaccharides. Results are presented as mean ± SEM. CCCP—carbonyl cyanide m-chlorophenylhydrazone; the sample indicates control for mitochondrial depolarization (cells grown in standard medium and treated with CCCP when stained with JC-1). Control—cells cultured in complete DMEM without XOS. XOS—cells grown for 24 h in complete DMEM containing 200 μg/mL XOS. * *p* < 0.05; ** *p* < 0.01.

JC-1 staining provides a suitable method for the assessment of the mitochondrial membrane potential state [37]. The dye forms aggregates in the functional mitochondria with active membrane potential or retains a monomeric form in the cytoplasm of cells with disrupted mitochondrial membrane potential. Hence, the JC-1 aggregate/monomer ratio indicates mitochondrial functionality and provides a tool to assess cellular health on a mitochondrial level. Figure 4B demonstrates the reduced mitochondrial membrane potential in XOS-treated cancer cells. JC-1 staining showed higher sensitivity for the XOS effect in cancer cells that preceded the effects on the level of ATP concentration (Figure 4). These results also support the MTT assay data that show reduced mitochondrial and metabolic activity of HT-29 and MRC-5 cells following longer treatment with XOS (Figure 3). Again, the nontumor MRC-5 fibroblasts were the least affected by the xylooligosaccharide sample. This fact supports the evidence for XOS antitumor activity.

### 2.3. XOS Reduce Proinflammatory Cytokines Production by U-937 Cells

**Figure 5:**
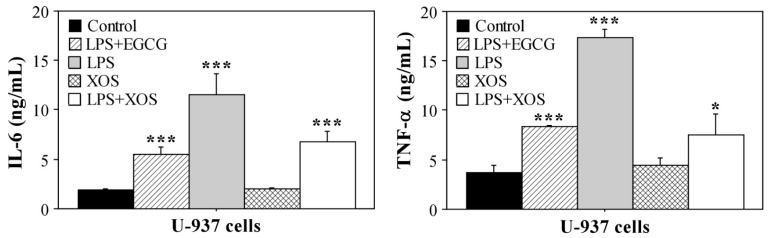
Concentrations of IL-6 and TNF-α in culture medium of U-937. Cytokine levels were measured by sandwich ELISA using culture medium of U-937 cells treated with XOS or LPS for 24 h. LPS+EGCG—cells treated for 1 h with 1 μg/mL EGCG followed by addition of 100 μg/mL LPS and culture for 24 h; LPS—U-937 cells stimulated with 100 μg/mL LPS for 24 h; XOS—cells grown for 24 h in culture medium containing 200 μg/mL XOS; LPS+XOS—cells treated for 1 h with 200 μg/mL XOS followed by addition of 100 μg/mL LPS and culture for 24 h. Results are shown as mean ± SEM. All samples were assayed in triplicates. Statistical significance was determined in comparison with the control (cells grown for 24 h in complete DMEM). * *p* < 0.05; *** *p* < 0.001.

The U-937 tumor cells demonstrated high sensitivity to XOS treatment. To further investigate this effect and determine its influence on the immune reactivity of U-937, we studied whether it persists in the presence of a stimulus that induces the production of proinflammatory cytokines by the cells. Lipopolysaccharide (LPS) was used to activate cytokine production by U-937 cells without the induction of differentiation to a macrophage or dendritic cell phenotype [38]. A control with an inflammatory response inhibitor epigallocatechin-3-gallate (EGCG) [39] and LPS was included in the experiment, as well as a standard control with untreated cells. In addition to the sample incubated in a medium containing 200 μg/mL XOS, cells cultured for the same period (24 h) with LPS and 200 μg/mL XOS but pretreated with XOS for 1 h were included. The aim was to assess the potential of XOS to inhibit the LPS-mediated inflammatory response of U-937. The concentrations of TNF-α and IL-6 in the culture medium at the end of the experiment are shown in Figure 5. XOS treatment did not activate cytokine production by U-937. Moreover, in the presence of LPS, the XOS sample was able to reduce the inflammatory response in a similar extent as the inhibitor EGCG. The levels of both types of cytokines were strongly reduced compared with the sample from cells cultured in a medium with LPS only. Therefore, our results suggest that XOS could influence Toll-like receptor 4 (TLR4) signaling, which mediates LPS-activated inflammatory responses. Consequently, a question of whether XOS are able to bind TLR4 and thus inhibit proinflammatory signals during LPS stimulation arose. To clarify this issue, molecular docking analyses were performed.

### 2.4. Molecular Modeling of XOS-TLR4 Interaction

**Figure 6:**
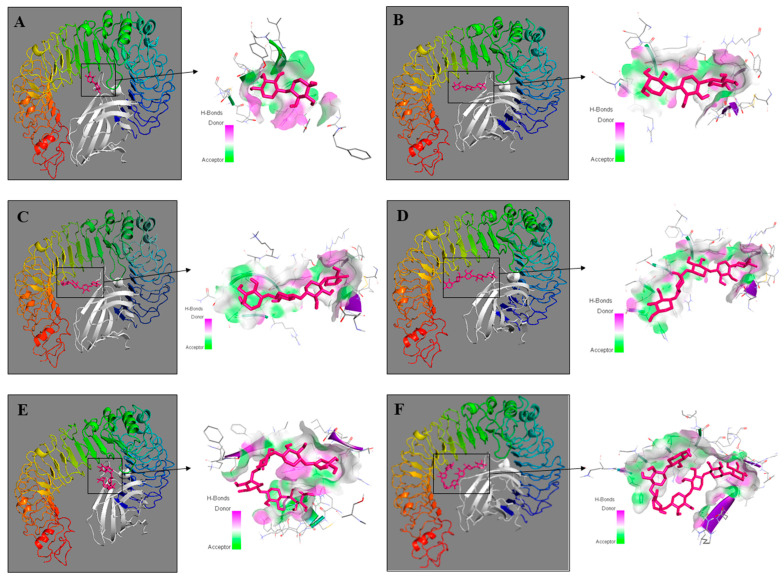
Three-dimensional structures of molecular docking studies representing binding affinity of xylooligosaccharides—xylobiose (**A**), xylotriose (**B**), xylotetraose (**C**), xylopentaose (**D**), xylohexaose (**E**), and xyloheptaose (**F**) (shown in pink) and TLR4 (chain A) and myeloid differentiation factor-2 (MD-2) (shown in rainbow and white, respectively). Protein–ligand complexes were visualized by Discovery Studio 2016 V16.1.0.

To assess the interaction between XOS and TLR4 (PDB ID: 3fxi), molecular docking was performed using Autodock vina in PyRx 0.8 software. Although the chemical analyses pointed out that the sample predominantly contained XOS with a DP of 6 or higher, molecular docking was performed for six xylose oligomers with DP ranging from 2 to 7 (xylobiose, xylotriose, xylotetraose, xylopentaose, xylohexaose, and xyloheptaose). The aim was to elucidate all XOS forms that could be present in the sample according to the manufacturers’ description. The best-docked complexes are displayed in Figure 6.

The results from the docking analyses showed that xylohexaose and xylopentaose bind to TLR4 with the highest binding affinity energy calculated as −8.3 kcal/mol and −8.1 kcal/mol, respectively. The binding affinity of xylotetraose (−7.7 kcal/mol) was almost equal to that of xyloheptaose (−7.6 kcal/mol). The docking studies of xylobiose and xylotriose with TLR4 showed only −6.4 kcal/mol and −6.7 kcal/mol, respectively (Table 3).

**Figure 7:**
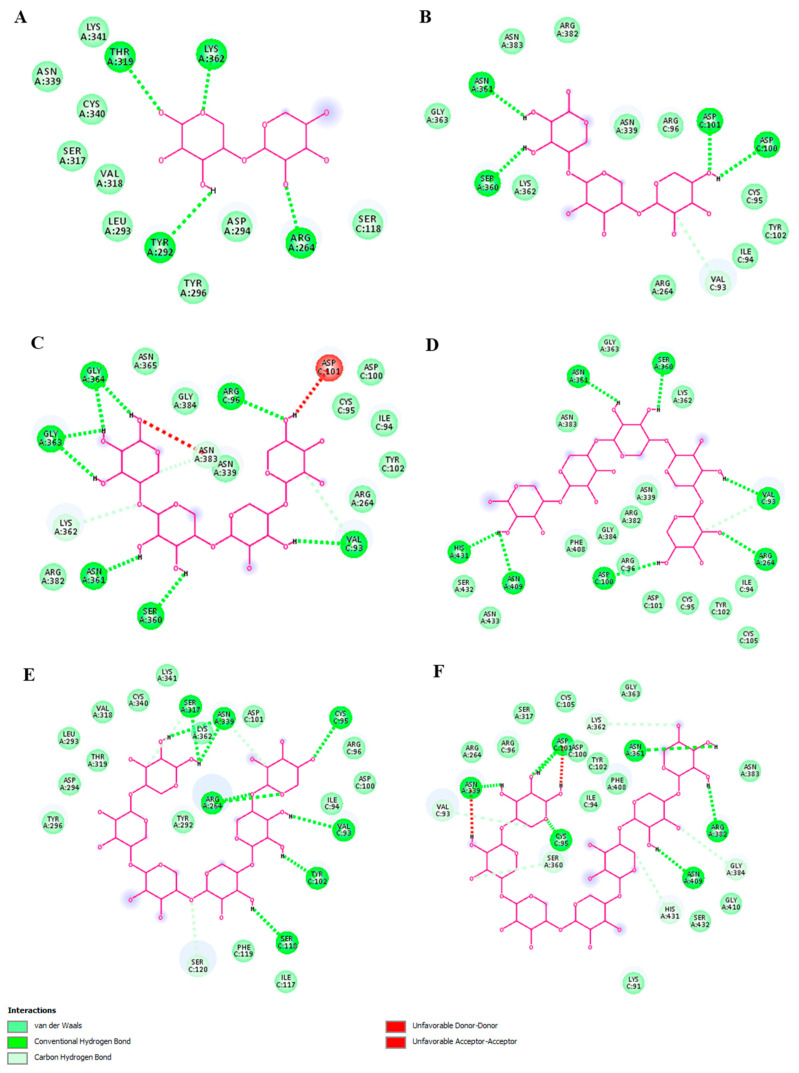
Two-dimensional diagrams of molecular docking studies representing binding affinity of xylooligosaccharides—xylobiose (**A**), xylotriose (**B**), xylotetraose (**C**), xylopentaose (**D**), xylohexaose (**E**), and xyloheptaose (**F**) and TLR4 (chain A) and myeloid differentiation factor-2 (MD-2). Diagrams were visualized by Discovery Studio 2016 V16.1.0.

The 2D visualization of the docking results showed that xylohexaose and xylopentaose formed conventional hydrogen bonds (H-bonds) with seven amino acid residues of TLR4 (chain A) and the coreceptor myeloid differentiation factor-2 (MD-2) (Figure 7D,E), xyloheptaose and xylotetraose formed H-bonds with six amino acid residues (Figure 7C,F), and xylobiose and xylotriose with four amino acid residues (Figure 7A,B). Xylotetraose interacted with the amino acid residue ASP101 with an unfavorable bond (Figure 7C). These results indicate that XOS with DP of 6, which is the predominant type in the studied sample, interact with TLR4 exhibiting the highest binding activity and ability to form H-bonds compared with XOS with DPs of 2, 3, 4, 5, or 7. Therefore, it could be suggested that the XOS sample could bind to TLR4 and influence the signaling pathways mediated by this receptor.

### 2.5. Xylooligosaccharide Treatment Affects Glutathione Homeostasis in Tumor Cells

The XOS sample demonstrated antioxidant potential, i.e., the ability to scavenge peroxyl and hydroxyl radicals. However, the sample showed a negative effect on the redox homeostasis particularly in tumor cells. A major antioxidant in all cell types is the reduced form of the tripeptide glutathione (GSH) [40]. During oxidative stress, GSH is converted to an oxidized form (GSSG). Therefore, glutathione levels and the GSH/GSSG ratio are important indicators for the cellular redox homeostasis and health status.

**Figure 8:**
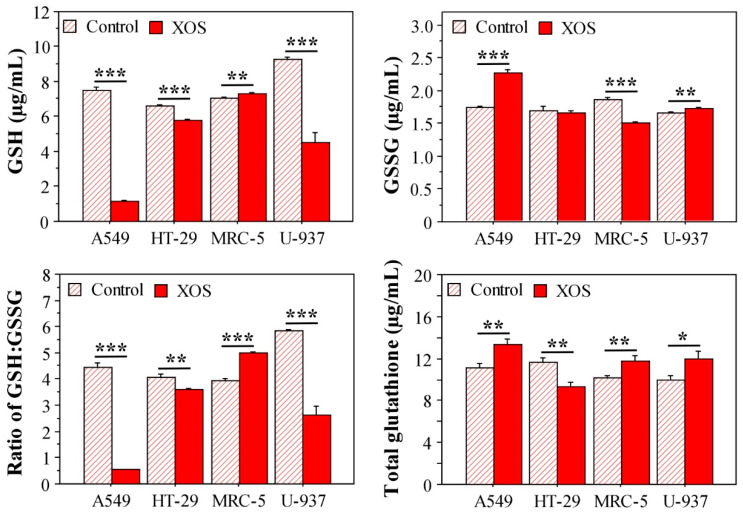
Glutathione levels in cells treated for 24 h with 200 μg/mL XOS. Results are displayed as ± SEM. All samples were assayed in triplicates. * *p* < 0.05; ** *p* < 0.01; *** *p* < 0.001.

To test if XOS influence the cellular redox state, we investigated the glutathione levels in A549, HT-29, MRC-5, and U-937 cells cultured in a medium containing 200 μg/mL of XOS for 24 h. The potential effect of XOS treatment on the intracellular concentrations of GSH and GSSG, as well as the GSH/GSSG ratio, and the total glutathione were analyzed. Results from these evaluations pointed to an interesting fact. As demonstrated in Figure 8, XOS-treated tumor cells contained reduced levels of GSH compared with the control ones that were cultured in a standard medium. Only MRC-5 cells did not show a difference in the GSH concentration. The oxidized glutathione form was reduced in XOS-treated MRC-5 cells, while this effect was not evident for the XOS-treated tumor cells. Conversely, A549 cells had increased GSSG after culture in the presence of XOS. The ratio GSH/GSSG illustrated these effects and even strengthened them. All tumor cell types treated with XOS had a reduced GSH/GSSG ratio, but MRC-5 fibroblasts showed an opposite result. These data highlight a tendency for the inability of tumor cells to convert oxidized glutathione to its reduced form when treated with XOS. Considering the elevated oxidative stress in tumor cells, aberrations in the process of glutathione reduction could have a detrimental effect on the redox balance and eventually lead to impaired cell functionality as demonstrated by the MTT and NR assays (Figure 3).

**Figure 9:**
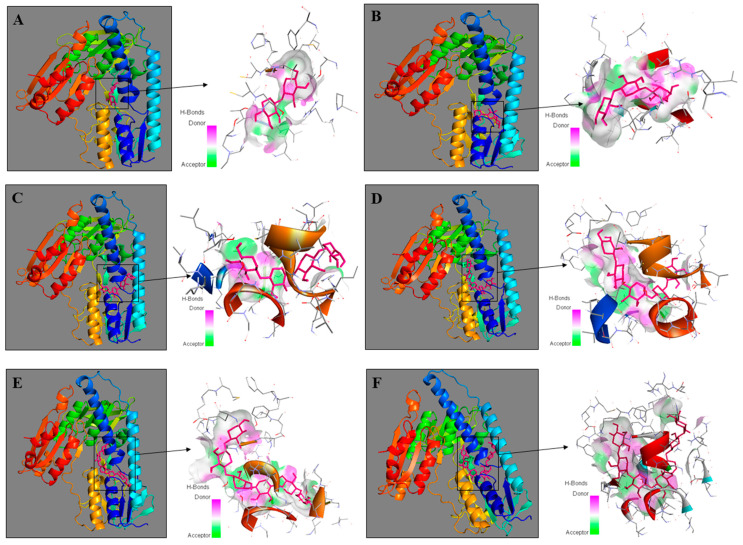
Molecular docking of xylooligosaccharides—xylobiose (**A**), xylotriose (**B**), xylotetraose (**C**), xylopentaose (**D**), xylohexaose (**E**), and xyloheptaose (**F**) (shown in pink) and human glutathione reductase (shown in rainbow). Protein–ligand complexes were visualized by Discovery Studio 2016 V16.1.0.

A key enzyme in the conversion of GSSG to GSH is glutathione reductase (GR) [41]. Based on the experimental results, we suggested a potential link between XOS and glutathione reductase activity. A direct interaction between these molecules cannot be excluded. Moreover, it is presumable based on the results for the GSH/GSSG ratio. To support this suggestion and determine the XOS potential for interaction with GR, molecular docking analyses were carried out. Again, all six XOS forms with DP from 2 to 7 were included in the evaluations. XOS protein docking was used to assess the interaction between xylobiose, xylotriose, xylotetraose, xylopentaose, xylohexaose, xyloheptaose, and the human glutathione reductase. The estimated binding energy and pose of interaction are two crucial factors in this analysis. The 3D structures of the best-docked complexes are illustrated in Figure 9.

**Figure 10:**
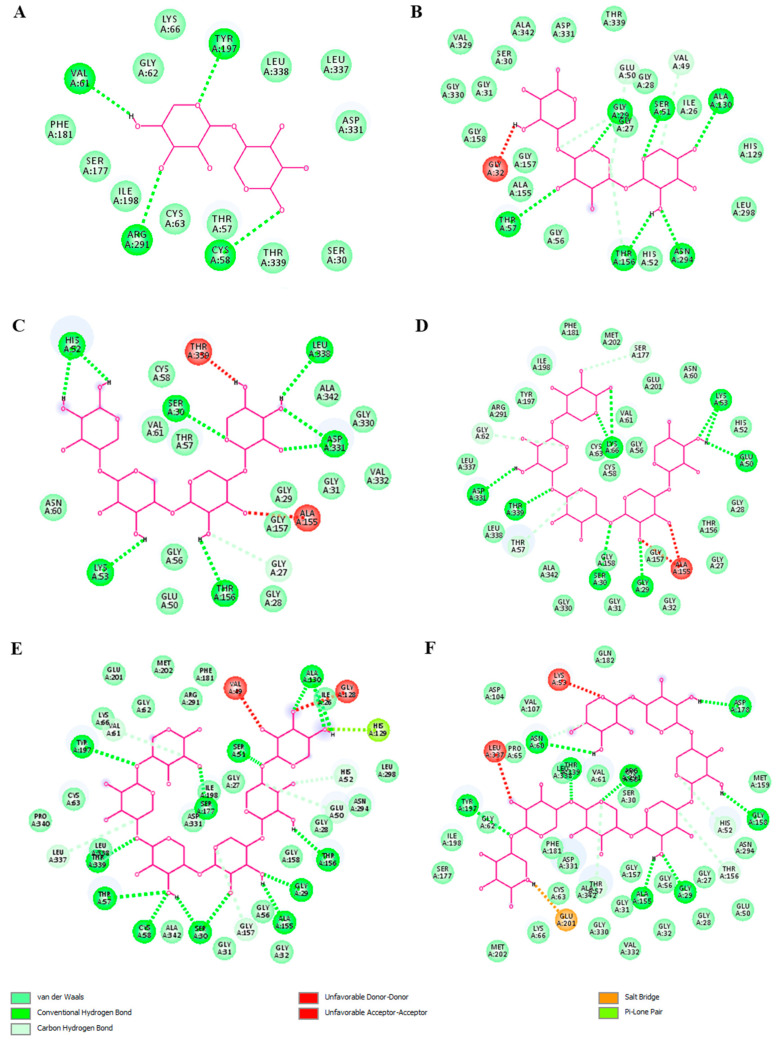
2D interaction poses of human glutathione reductase with xylooligosaccharides—xylobiose (**A**), xylotriose (**B**), xylotetraose (**C**), xylopentaose (**D**), xylohexaose (**E**), and xyloheptaose (**F**). Diagrams were visualized by Discovery Studio 2016 V16.1.0.

The xylooligosaccharides with DP 5, 6, and 7 had the best binding affinity energy scores of −11.0, −11.7, and −11.9 kcal/mol, respectively. Xylotriose and xylotetraose had the same binding energy (−9.7 kcal/mol). The docking study of xylobiose and the human glutathione reductase showed only −7.7 kcal/mol binding energy (Table 3). The XOS formed strong and stable binding with the human GR regarding conventional hydrogen bonds (Figure 10). Hydrogen bonds are the prevailing intermolecular interactions in biological complexes and significantly contribute to the specificity of molecular recognition [42]. The XOS formed between 4 (xylobiose) and 11 (xylohexaose) conventional hydrogen bonds. Xylotriose, xylotetraose, xylopentaose, xylohexaose, and xyloheptaose interacted with one or two amino acid residues with an unfavorable bond (Figure 10). Xylohexaose and xyloheptaose were determined to form the highest number of conventional hydrogen and carbon hydrogen bonds with glutathione reductase suggesting a strong capacity to interact with the protein. Hence, it can be considered that these data support our hypothesis based on the data for glutathione levels in XOS-treated tumor cells. The tested oligosaccharide sample predominantly contained XOS with DP 6 and also XOS with a higher DP (7), which were shown by the docking analyses as the best binders to glutathione reductase.

## 3. Discussion

Xylooligosaccharides have been shown to manifest excellent prebiotic properties [9,16], but these compounds also possess other important biological activities that could be beneficial for human health. They include antitumor, antimicrobial, immunomodulatory, and anti-inflammatory properties [15]. Particularly, the present study focused on one of them—the antitumor potential specifically of a commercial XOS natural product. Reasonably, the biological evaluations of the sample were preceded by detailed chemical characterization of the XOS sample and analysis of its antioxidant activity. They provide important information that supplements and favors better interpretation of biological data. In support of this statement, it has been demonstrated that the antioxidant properties of the XOS sample that mainly contained xylobiose contributed to its preventive effect against colorectal cancer [30].

It is known that XOS are composed of *β*-1,4-linked d-xylose residues with a low DP between 2 and 10 [11] or up to 20 units [1]. The low uronic acid content in the analyzed sample might be a result of the preparation of XOS by an acidic hydrolysis of heteroxylans, which usually contain small quantities of (4-*O*-methyl-) α-d-glucuronic acid [4,11]. XOS also contained representative amounts of glucose. In general, different low-cost lignocellulosic materials are preferred for the enzymatic or chemical production of XOS, such as corn cob, wheat straw, and sugar cane bagasse, and the glucose present can be also a result of partly cellulose degradation during the preparation of XOS [11,43]. Xyl residues can be acetylated at *O*-2 and/or *O*-3 in heteroxylans, as acetyl esters are important for the chemical interactions in the cell wall matrix and plant physiology, and they also inhibit the enzyme and chemical degradation of xylans into XOS [4,5]. The estimated acetyl content (0.65%) in the studied XOS is much lower than that in xylans from bagasse (8.7%) and straw (2.4%), which was expected considering the technology for the production of XOS [44]. Apart from that, the influence of acetylation on the biological activity of XOS is still unknown.

The XOS sample expressed in vitro antioxidant activity through the ORAC and HORAC methods. Interestingly, Boonchuay et al. (2021) determined high DPPH, ABTS, and TRAP antioxidant activities of corn cob XOS; however, their sample also contained a high total phenolic content (0.32 mg gallic acid equivalents (GAE)/mg), which makes impossible the determination of the antioxidant potential of the XOS [14]. Similarly, Ávila et al. (2020) demonstrated that XOS prepared from sugarcane straw xylan expressed a dose-dependent total antioxidant capacity between 0.053 and 0.8 g/L and a DPPH radical-scavenging activity of 71% at a concentration of 2 g/L [31]. Data for comparative analysis were found for the ORAC test only. Zhang et al. (2019) prepared spray-dried XOS (40% *w/v*) carried by gum Arabic (5% *w/v*), which expressed 71.9 ± 1.1 and 61.8 ± 0.4 μmol Trolox equivalents (TE)/g ORAC activity before and after drying, respectively [45]. This activity was much lower than the value determined in our study, which can be explained by the differences in the XOS origin, preparation, and DP. According to the results of the HPSEC-RID analysis, our sample contained considerable amounts of XOS with DP ≥ 6, whereas their sample contained about 92% XOS with DP between 2 and 4. It has also been found that the antioxidant activity of acidic XOS from corn-cob-derived xylan was involved in its inhibitory activity against stress-induced gastric inflammation in mice [46]. Furthermore, supplementation of diabetic Wistar rats with XOS for 6 weeks reduced severe glucosuria, proteinuria, blood creatinine, and urea levels, formation of advanced glycation end products; and elevated antioxidant enzyme activities [47]. Therefore, it can be suggested that the antioxidant activity of XOS might be useful in the supplementary therapy of oxidative stress-related diseases.

The present study demonstrated an antitumor activity of the analyzed XOS sample. In vitro cytotoxicity assays indicated that XOS affected to a lesser extent MRC-5 cells that were derived from nontumor tissue, but the three tested tumor cell lines showed time- and concentration-dependent inhibition. Higher cytotoxicity levels in tumor cells following exposition to XOS were determined via the NR uptake assay, which highlighted the intracellular inhibitory effects on the lysosome level. Mitochondrial functionality was also affected as detected by MTT assays, ATP measurements, and mitochondrial membrane potential analyses. A recent article by Ghosh et al. reported the inhibitory effects of a mixed XOS sample on HT-29 and Caco-2 colon adenocarcinoma cells detected by MTT assays, which were more pronounced compared with our results [28]. Similar inhibitory potential was determined in our study via NR assays suggesting a different mechanism of action of the analyzed XOS. A possible reason for this could be the composition and complexity of the studied samples. The XOS in the report of Ghosh et al. contained a mixture of xylobiose, xylotriose, and xylotetraose derived from sugarcane bagasse, while the XOS in the present study were with higher DP (6 and 7). Other research groups have also shown that the antitumor activity of XOS with different DP. XOS isolated from green algae with DP of about 5 inhibited the proliferation and induced the apoptosis of breast cancer MCF-7 cells [27]. Ando et al. detected reduced cell viability and ATP production of acute lymphoblastic leukemia cells (Jurkat and MOLT-4) after treatment with bamboo-derived XOS fraction with DP of 2–15 [26]. Analogously, the lymphoma cell line U-937 included in our experiments showed high sensitivity to XOS treatment, which was detected on the level of ATP production, mitochondrial membrane potential, cell viability, and lysosome activity. Moreover, XOS inhibited the LPS-mediated production of proinflammatory cytokines by U-937 cells to an extent similar to the effect of EGCG, which has been shown to inhibit TLR4 activation and signaling [39]. Therefore, our results indicate that the antitumor effect of XOS can involve interaction with TLR4. The carbohydrate components of LPS have an important role for the interaction with TLR4 and its coreceptor MD-2 leading to the activation of proinflammatory signaling pathways [48]. Thus, XOS binding to TLR4 and the interaction inducing the inhibition of downstream signaling cannot be excluded. In fact, results from the docking analyses confirmed this hypothesis showing that xylohexaose could interact with TLR4 and MD-2 with the best binding affinity compared with XOS with DPs of 2, 3, 4, 5, and 7. Our results point to an inhibitory interaction between XOS and TLR4 that prevents proinflammatory response and possibly influences cell viability. However, it cannot be excluded that XOS binding to MD-2 and TLR4 may induce conformational changes that induce endocytosis and lead to cellular internalization and lysosomal localization of the ligand–receptor complex where XOS can dissociate and interact with different intracellular targets. This speculation is motivated by the strong lysosome-specific effect detected by the NR uptake assays in our experiment. However, future experiments investigating TLR4 internalization and intracellular localization after XOS treatment can confirm this hypothesis and clarify the mechanism of this putative effect.

TLR4 has been associated with cancer progression [49,50] and shown to be overexpressed or aberrantly expressed in breast, colon, ovarian, liver, and other cancer cell types [50]; however, to date, a direct link between the XOS antitumor effect and TLR4 or an interaction between XOS and TLR4 has not been reported. So far, XOS antitumor effects have been attributed to the modulation of gut microbiota and activation of short-chain fatty acid (SCFA) production that were shown to be beneficial in hepatocellular carcinoma and colon cancer [29,49]. Interestingly, in line with our findings, a recent study demonstrated an interaction between TLRs and other prebiotic carbohydrates—*β*-(2→6)-type fructans [51]. Moreover, it was proved that Graminan-type fructans strongly inhibit TLR4 and attenuate proinflammatory responses [51]. We suggest that XOS interaction with TLR4 has similar effects based on the detected reduction in proinflammatory cytokine production by LPS-stimulated U-937, and this inhibition further contributes to the antitumor activity of the sample. Indeed, another recent study identified the Arg241 residue of TLR4 and the Tyr102, Ser120, and Lys122 residues of MD-2 as the key binding sites of antagonistic ligands [52]. These data strongly support our results because docking analyses of the XOS-TLR4 and MD-2 interaction showed the ability of xylohexaose to bind at position Tyr102 in MD-2 and strengthen our hypothesis for the inhibitory effect of the XOS sample on TLR4 signaling. In addition, all tumor cell lines included in our experiments have been shown to express TLR4 [38,53,54]—a fact that supports the connection between the detected antitumor effects and XOS–TLR4 interaction.

Apart from the hypothesized TLR4-mediated endocytosis, XOS can enter cells via plasma membrane transporters and influence specific intracellular targets including the glutathione antioxidant system, which is a major regulator of redox state in normal and cancer cells [41]. The present report demonstrated that XOS influence the GSH/GSSG ratio by reducing the intracellular concentration of GSH, which is of critical importance for the survival and development of tumor cells that exhibit elevated levels of oxidative stress due to their high metabolic rate and activation of ROS-coupled signaling pathways [40]. In fact, a number of strategies for cancer treatment have been developed aiming to manipulate the glutathione homeostasis by reducing GSH synthesis, increasing GSSG levels, and, thus, reducing the GSH/GSSG ratio [40]. Therefore, XOS treatment represents another mode of action that contributes to its antitumor potential. This tumor-specific influence on GSH homeostasis led us to propose that XOS can influence the activity of key enzymes involved in maintaining the cellular redox state. In relation to the diminished GSH/GSSG ratio, glutathione reductase has the major role for the conversion of GSS to GSH. Drug-resistant tumor cells have been shown to express high levels of GR, and depletion and inhibition of GR restore their susceptibility to therapeutic agents [55]. Intriguingly, our molecular docking analyses showed that XOS can interact with GR, and based on the detected reduced GSH/GSSG ratio in tumor cells, we propose that this interaction leads to enzyme inhibitory effect. Future experiments are needed to clarify in detail this property of XOS antitumor activity.

## 4. Materials and Methods

### 4.1. Xylooligosaccharides

XOS isolated from corn were purchased from Lyphar Biotech Co., Ltd. (Xi′an, China) and supplied in a lyophilized form. For biological evaluations, the xylooligosaccharides were dissolved in sterile Dulbecco’s modified phosphate buffered saline (DPBS) to yield a stock solution with a concentration of 2 mg/mL, which was passed through a sterile 0.2 μm membrane syringe filter (Corning Inc., Glendale, AZ, USA) and kept at 4 °C until use.

### 4.2. General Chemical Analyses

The total carbohydrate content in the XOS sample was determined by the colorimetric phenol-sulfuric acid method, as described by DuBois et al. (1956) [56], using xylose as a standard. Absorbance was measured at 480 nm. The total uronic acid content was assayed in a Skalar San++ autoanalyzer (Analytical BV, Breda, The Netherlands) according to the manufacturer′s instructions and using the m-hydroxydiphenyl method of Blumenkrantz and Asboe-Hansen (1973) [57]. Glucuronic acid was used as a calibration standard. Control samples without the addition of chromogen were prepared for the correction of the results for a nonspecific color formation during the sulfuric acid treatment. The total phenolic content was analyzed using the method of Singleton and Rossi (1965) with the Folin–Ciocalteu reagent [58]. Gallic acid served as a standard. The acetyl content was determined according to the colorimetric hydroxamic acid reaction using *β*-d-glucose pentaacetate as a standard [59]. The qualitative iodine test for the detection of starch in XOS (200 mg/mL) was performed with Lugol′s solution, containing 5% iodine and 10% KI. Commercial potato starch (Merck KGaA, Darmstadt, Germany) was used as a positive control. The XOS sample was analyzed in a triplicate, and the results from the three analyses were expressed in percent (*w/w*) ± standard deviation (SD).

### 4.3. Monosaccharide Composition Analysis

The XOS sample (10 mg) was separately hydrolyzed with 0.2 and 2 M trifluoroacetic acid (TFA) (5 mL) for 70 min at 120 °C in a block heater SBH200D (Stuart^®^, Stone, Staffordshire, UK). The resulting hydrolysates were evaporated to dryness at 40 °C under reduced pressure and redissolved in 10 mL ultrapure water. For a complete release of TFA, the samples were additionally evaporated twice. Then, the hydrolysates were dissolved in 1 mL ultrapure water and centrifuged at 18,187× *g* at 20 °C for 10 min. Finally, the samples were analyzed on a Nexera-i LC2040C Plus UHPLC system (Shimadzu, Tokyo, Japan) equipped with a Zorbax Carbohydrate column (4.6 × 150 mm, 5 μm, Agilent Technologies Inc., Santa Clara, CA, USA) and a precolumn Zorbax Reliance Cartridge. The sample injection volume was 10 μL, and an ultrapure water–acetonitrile mixture (20:80, *v/v*), in an isocratic regime, served as a mobile phase. The column temperature was 35 °C, and a flow rate of 0.6 mL/min was applied. The eluate was monitored using a refractive index detector RID-20A, operating at 40 °C. Data were acquired using LabSolutions DB software (Shimadzu, Tokyo, Japan). Monosaccharides were identified by comparing the retention times of unknown analytes with analytical-grade standards (Glc, Man, Fru, Gal, Rha, Xyl, Fuc, and Ara) delivered by Merck KGaA (Darmstadt, Germany). The results were calculated from the relationship between the peak area response and concentration (0.5–10 mg/mL) using linear regression for each analyte. The R-squared values (R^2^) were > 0.999 for all calibration curves. The sample was analyzed in a duplicate, and the results were expressed in percent (*w/w*) ± SD (*n* = 3).

### 4.4. Molecular Weight Distribution Analysis

The molecular weight distribution of the XOS sample was analyzed using a Nexera-i LC2040C Plus UHPLC system (Shimadzu, Tokyo, Japan) equipped with a Bio SEC-3 column (4.6 × 300 mm, 300 Å, 3 μm, Agilent Technologies Inc., Santa Clara, CA, USA) and a refractive index detector RID-20A. The sample injection volume was 10 μL and as a mobile phase served 150 mM NaH_2_PO_4_ (pH = 7.0) at a flow rate of 0.5 mL/min. The column and detector temperatures were 30 °C and 40 °C, respectively. The sample (10 mg/mL) was centrifuged at 18,187× *g* for 10 min at 20 °C prior to analysis. Data were acquired using LabSolutions DB software (Shimadzu, Tokyo, Japan). The elution profile of the XOS sample was compared with the profiles of pullulan standards (2 mg/mL, 0.59 × 104–78.8 × 10^4^ g/mol, Shodex^®^ standard P-82 kit, Showa Denko-K.K., Tokyo, Japan) and the following oligosaccharides (2 mg/mL): laminaritriose, laminaritetraose, laminaripentaose, and 1,3:1,6-*β*-d-glucohexaose (Megazyme Inc., Bray, Ireland).

### 4.5. Fourier Transform Infrared Spectroscopy

The FTIR spectrum of XOS was obtained in the region of 500–4000 cm^−1^ using the ATR technique on a Tenzor 27 (Bruker GmbH, Leipzig, Germany), controlled by OPUS 8.7. software. The FTIR spectrum was analyzed in Spectragryph software (Friedrich Menges, Oberstdorf, Germany).

### 4.6. In Vitro Antioxidant Activity

ORAC activity was analyzed according to the method of Ou et al. [60], with some modifications described in detail by Teneva et al. [61]. The analysis was performed at 37 °C on a fluorometer FLUOstar OPTIMA (BMG Labtech, Offenburg, Germany) with an excitation wavelength of 485 nm and emission wavelength of 520 nm. Trolox (Merck KGaA, Darmstadt, Germany) was used for the construction of a standard curve. The sample was analyzed in a duplicate, and the results were expressed in μmol Trolox equivalents (TE) per gram of sample ± SD (*n* = 6). HORAC activity was determined according to Ou et al. [62], as described in detail by Teneva et al. [61]. The analysis was performed at 37 °C on a fluorometer FLUOstar OPTIMA (BMG Labtech, Offenburg, Germany) with an excitation wavelength of 485 nm and emission wavelength of 520 nm. Gallic acid was used as a calibration standard. The sample was analyzed in a duplicate, and the results were expressed in micromole gallic acid equivalents (μmol GAE) per gram of sample ± SD (*n* = 6).

### 4.7. Cell Lines and In Vitro Culture Conditions

The following human cell lines were used in the present study: A549 (lung carcinoma origin; ATCC^®^ CCL-185^TM^), HT-29 (derived from colon adenocarcinoma, ATCC^®^ HTB-38^TM^), MRC-5 (normal lung fibroblasts; ATCC^®^ CCL-171^TM^), and U-937 (histiocytic lymphoma origin, ATCC^®^ CRL-1593.2^TM^). The cells were cultured in a complete growth medium, i.e., Dulbecco’s modified Eagle’s medium (DMEM) (Merck KGaA, Darmstadt, Germany) supplemented with 10% heat-inactivated fetal bovine serum (FBS) (Merck KGaA, Darmstadt, Germany), 100 U/mL penicillin, and 100 μg/mL streptomycin (Merck KGaA, Darmstadt, Germany). All cell lines were manipulated under sterile conditions and maintained in a humidified incubator at 37 °C and 95% atmospheric air/5% CO_2_ content. Prior to the experiments, the cultures were expanded in 75 cm^2^ flasks (TPP, Trasadingen, Switzerland) up to 80% confluency. Then, the cells were trypsinized; the concentration of viable cells was determined and adjusted to 1 × 10^5^ cells/mL.

### 4.8. In Vitro Cytotoxicity and Antitumor Activity Assays

The prepared cell suspensions (1 × 10^5^ cells/mL; 200 μL/well) were plated on 96-well plates (TPP, Trasadingen, Switzerland) and cultured for 24 h under standard conditions (37 °C, 5% CO_2_ content, and high humidity). Then, the cells were incubated in a complete growth medium containing various XOS concentrations (50, 100, and 200 μg/mL) for three different test periods—24, 48, and 72 h. The sterile XOS sample was directly added and diluted in the culture medium. Untreated cells cultured for the same test periods in complete DMEM served as a negative control. Cells treated with 100 μg/mL mitomycin C (Merck KGaA, Darmstadt, Germany) were used as a positive control.

The cytotoxicity of the XOS sample was analyzed by MTT and NR assays. The MTT tests were performed in accordance to the methodology described by Edmondson et al. [63]. At the end of every test period, 5 mg/mL MTT (Merck KGaA, Darmstadt, Germany) solution was added to all culture wells. The reagent was directly diluted in the culture medium yielding a final concentration of 0.5 mg/mL. Then, the cells were incubated for 2 h in a humidified incubator at 37 °C and 5% CO_2_–atmospheric air content. After that, the amount of accumulated formazan formed by MTT reduction in viable cells was determined. The plates with U-937 cells were centrifuged, and the MTT-containing medium in all test plates was removed. Then, 100 μL DMSO was added to each test well. The cells were incubated for 15 min at room temperature on a shaker, and then absorbance at 570 nm wavelength was measured on a Synergy-2 reader (BioTek, Winooski, VT, USA).

Neutral red uptake assays were performed in accordance to the method described by Repetto et al. [64]. NR staining enables the evaluation of lysosome functionality and cell viability following treatment with a test agent because the dye accumulates in the lysosomal matrix of viable cells. In our experiments, A549, HT-29, MRC-5, and U-937 cells were expanded, detached, seeded on 96-well plates (TPP, Trasadingen, Switzerland), and treated with XOS using the same methodology as for the MTT assay. After 24, 48, and 72 h culture in a medium containing different XOS concentrations, NR solution (Merck KGaA, Darmstadt, Germany) was added to the cells to a final concentration of 0.5 mg/mL. The cultures were stained for 2 h at 37 °C and high humidity. Then, the plates with U-937 cells were centrifuged, the culture medium of all test plates was aspirated, and 100 μL solution containing 50% ethanol and 1% acetic acid was added to all samples in order to extract the stain accumulated in the cells. Following 15 min incubation under continuous mild shaking, the absorbance at 540 nm was measured using a Synergy-2 reader (BioTek, Winooski, VT, USA). Results from both in vitro cytotoxicity assays were expressed as the percent inhibition of cell viability and metabolic activity and lysosomal functionality based on the absorbance units from each test sample and the control cells incubated in a standard culture medium without XOS. The IC_50_ values for 72 h treatment with XOS were calculated. To determine the IC_50_ concentration for MRC-5 cells, triplicates treated with 400 μg/mL of XOS were assayed. Selectivity indexes for all tumor cell lines were defined based on the ratio IC_50_ normal cells/IC_50_ tumor cells. All samples were assayed in triplicates.

### 4.9. Evaluation of Cytokine Levels

To evaluate the proinflammatory cytokine production, U-937 cells (1.10^5^ cells/mL; 200 μL/well) were seeded on 96-well plates (TPP, Trasadingen, Switzerland). The following samples were set in triplicates and cultured under standard conditions for 24 h: untreated U-937 grown in a complete growth medium (control); U-937 cultured in a medium containing 100 μg/mL LPS (Sigma-Aldrich, Merck KGaA, Darmstadt, Germany); cells grown in a medium supplemented with 200 μg/mL XOS; U-937 cultured for 1 h with 1 μg/mL epigallocatechin 3-gallate (Calbiochem, Merck KGaA, Darmstadt, Germany) followed by the addition of 100 μg/mL LPS; and U-937 cultured for 1 h with 200 μg/mL XOS followed by the addition of 100 μg/mL LPS. At the end of the 24 h incubation period, a 120 μL culture medium from all test wells was collected and used for enzyme-linked immunosorbent assays to determine IL-6 and TNF-α levels. The concentration of cytokines in the cell culture medium was measured using human IL-6 and TNF-α ELISA kits (ImmunoTools GmbH, Friesoythe, Germany) according to the manufacturers’ protocol. All samples were analyzed in triplicates. The components of the cell culture medium did not show immunoreactivity in the cytokine ELISAs.

### 4.10. ATP Determination

For the measurement of intracellular ATP concentration, A549, HT-29, MRC-5, and U-937 cells were seeded on 6-well plates (TPP, Trasadingen, Switzerland) (0.5 × 10^6^ cells/well) and incubated under standard conditions for 24 h. Subsequently, 200 μg/mL XOS were added to duplicate wells of each cell line. Duplicate wells with cells cultured for the same time in complete DMEM served as positive control. At the end of the 24 h treatment, A549, HT-29, and MRC-5 cells were detached, and U-937 cells were resuspended. Then all cell samples were transferred to Eppendorf tubes, washed with sterile DPBS, and lysed. A negative control with nonlysed cells was set for each cell type. The ATP concentration was measured using a standard ATP Determination Kit A22066 (Invitrogen^TM^, Thermo Fisher Scientific Inc., Waltham, MA, USA)) according to the manufacturer’s protocol. Detection of ATP was based on a bioluminescent reaction with D-luciferin catalyzed by the enzyme luciferase [65]. The reaction is extremely sensitive and allows the detection of ATP in a concentration of up to 0.1 picomole. Bioluminescence measurements were carried out on a GloMax^®^ 20/20 luminometer (Promega Corporation, Madison, WI, USA).

### 4.11. JC-1 Staining

A 5,5′,6,6′-tetrachloro-1,1′,3,3′-tetraethylbenzimidazol-carbocyanine iodide /JC-1/ (eBioscienceTM, Thermo Fisher Scientific Inc., Waltham, MA, USA) dye was used to assess the extent of cells with impaired mitochondrial membrane potential following XOS treatment. JC-1 accumulates in the cytoplasm of the cell in the form of monomers with absorption and emission maximum of approximately 485 ± 20 and 528 ± 20 nm, respectively. In cells with an active mitochondrial membrane potential, JC-1 forms aggregates with absorption and emission spectrum of 540 ± 25 and 620 ± 40 nm, respectively, which specifically stain the functional mitochondria in the cell. The appearance of red fluorescence in JC-1 staining solely depends on the mitochondrial membrane potential [37]. In our experiments, cells from the A549, HT-29, MRC-5, and U-937 cell lines were seeded at a concentration of 1 × 10^5^ cells/mL on 96-well culture plates and incubated at 37 °C for a period of 24 h, after which the cells were treated with 200 μg/mL XOS for 24 h. Control cells cultured for the same period in complete DMEM were included in the experiment for each cell line. Before staining, the cells were washed once with prewarmed DPBS buffer containing 5% fetal calf serum. Then, JC-1 in 2 μM final concentration was added to the culture wells followed by incubation at 37 °C and 5% CO_2_ for 15–30 min in the dark. For mitochondrial depolarization positive controls, CCCP in a final concentration of 50 μM was added and incubated with the cells at 37 °C for 5 min. Then, the cells were washed twice with prewarmed DPBS, and the fluorescence at 528 ± 20 nm (JC-1 monomer) and 620 ± 40 nm (JC-1 aggregate) was measured using a SpectraMax i3x microplate reader (Molecular devices, San Jose, CA, USA). All samples were analyzed in triplicates.

### 4.12. Molecular Docking

The interaction performance between the XOS (xylobiose, xylotriose, xylotetraose, xylopentaose, xylohexaose, and xyloheptaose) and the TLR4 were evaluated through molecular docking using Autodock vina in PyRx 0.8 [66]. The three-dimensional structures of the XOS xylobiose (PDB ID: 3w25), xylotriose (PDB ID: 4pvi), xylotetraose (PDB ID: 2y6k), xylopentaose (PDB ID: 1uxx), xylohexaose (PDB ID: 4prw), and xyloheptaose (PDB ID: 5ofk) and the TLR4 (PDB ID: 3fxi) were downloaded from the RCSB Protein Data Bank (http://www.rcsb.org/pdb/home/home.do, accessed on 9 May 2022) in PDB format [67]. The retrieved crystal structures were in a complex form of protein and ligand; hence, Discovery Studio 2016 V16.1.0 was used to separate the protein and ligand from the complex structures [68]. All water molecules, heteroatoms, hydrogens, and Gasteiger–Marsili charges in the retrieved target file 3fxi were removed. The three-dimensional coordinates of all the ligand structures were prepared using UCSF Chimera (v. 1.14) [69]. The energy form of the ligands was minimized. The files were then converted into pdbqt format (suitable for docking with AutoDock Vina) using OpenBabel [70]. The docking algorithms were set to default. The size of the grid box in AutoDock Vina was kept at 34.158 × 38.564 × 47.750 Å for X-, Y-, and Z-axes, respectively. The best-docked complex was selected based on the lowest energy scoring in kcal/mol and docking efficiency.

Docking simulations between the XOS (xylobiose, xylotriose, xylotetraose, xylopentaose, xylohexaose, and xyloheptaose) and the human glutathione reductase (EC 1.8.1.7) were performed in the same way. The three-dimensional structure of the human GR was retrieved from the RCSB Protein Data Bank (PDB ID: 1bwc).

### 4.13. Assessment of Glutathione Levels

The intracellular concentrations of reduced (L-*γ*-glutamyl-L-cysteinylglycine), oxidized (GSSG; glutathione disulfide), and total glutathione were evaluated using a GSH, GSSG, and total glutathione determination kit (Abcam, Cambridge, UK). Cells treated with 200 μg/mL XOS and controls grown in a complete medium without XOS for 24 h were used in the experiment. To determine GSH, GSSG, and total glutathione concentrations, the protocol supplied by the manufacturer of the kit was strictly followed. Results were obtained by fluorescent intensity quantification of the samples using a SpectraMax i3x spectrophotometer (Molecular devices, San Jose, CA, USA) at Excitation/Emission ¼ 350/470 nm. All samples were assayed in triplicates.

### 4.14. Statistical Analysis

Statistical analyses were performed by one-way analysis of variance (ANOVA) and unpaired *t*-test using StatView Software version 5.0 (SAS Institute Inc., Cary, NC, USA). Data were considered significant when *p*  <  0.05.

## 5. Conclusions

The present report revealed a novel dual mode of antitumor action of commercially available xylooligosaccharides. We showed that XOS exert their antitumor effects by modulating TLR4 signaling and cellular redox state, which is affected by glutathione homeostasis. These important biological properties of XOS can be utilized to develop improved anticancer therapeutics and functional foods.

## Figures and Tables

**Table 1:** Chemical characterization and in vitro antioxidant activity of xylooligosaccharides.

Parameters	Values
Total carbohydrate content [%, *w/w*]	97.3 ± 2.4
Total uronic acid content [%, *w/w*]	<1
**Monosaccharide composition [%, *w/w*]**	
Xylose	59.4 ± 2.9
Degree of acetylation [mol%] ^1^ (Acetyl content [%, *w/w*])	3.3 ± 0.1 (0.6 ± 0.01)
Glucose	9.7 ± 1.1
Total proteins [%, *w/w*]	n.f. ^2^
Total phenolics [%, *w/w*]	0.2 ± 0.01
ORAC ^3^ [μmol TE ^4^/g]	1150.2 ± 32.8
HORAC ^5^ [μmol GAE ^6^/g]	303.1 ± 9.7

^1^ Moles of acetyl groups per 100 moles of xylose; ^2^ not found; ^3^ oxygen radical absorbance capacity; ^4^ Trolox equivalents; ^5^ hydroxyl radical averting capacity; ^6^ gallic acid equivalents.

**Table 2:** IC_50_ and selectivity index values of XOS for different cell lines ^§^.

	A549	HT-29	U-937	MRC-5
Mean IC_50_ (μ g/mL)	143.3 ± 7.1 ***	51.8 ± 0.4 ***	150 ± 9.6 ***	367.3 ± 9.3
Selectivity index	2.6	7.1	2.5	NA

^§^ All values were determined using NR assay data for 72 h XOS treatment. IC_50_ values are presented as mean ± standard error of the mean (SEM). Asterisks (*) indicate statistical significance. *** *p* < 0.001; NA—not applicable.

**Table 3:** Binding affinity of xylooligosaccharides to Toll-like receptor 4 (TLR4) and to human glutathione reductase (huGR).

	Xylobiose (kcal/mol)	Xylotriose (kcal/mol)	Xylotetraose (kcal/mol)	Xylopentaose (kcal/mol)	Xylohexaose (kcal/mol)	Xyloheptaose (kcal/mol)
TLR4	−6.4	−6.7	−7.37	−8.1	−8.3	−7.6
huGR	−7.7	−9.7	−9.7	−11.0	−11.7	−11.9

## Data Availability

Data are contained within the article or available from the corresponding author upon request.

## References

[1] Moure A., Gullón P., Domínguez H., Parajó J.C. (2006). Advances in the manufacture, purification and applications of xylo-oligosaccharides as food additives and nutraceuticals. Process Biochem..

[2] Wierzbicki M.P., Maloney V., Mizrachi E., Myburg A.A. (2019). Xylan in the Middle: Understanding Xylan Biosynthesis and Its Metabolic Dependencies Toward Improving Wood Fiber for Industrial Processing. Front. Plant Sci..

[3] De Freitas C., Carmona E., Brienzo M. (2019). Xylooligosaccharides production process from lignocellulosic biomass and bioactive effects. Bioact. Carbohydr. Diet. Fibre.

[4] Ebringerová A., Hromádková Z., Heinze T., Heinze T. (2005). Hemicellulose. Polysaccharides I. Structure, Characterization and Use.

[5] Santibanez L., Henriquez C., Corro-Tejeda R., Bernal S., Armijo B., Salazar O. (2021). Xylooligosaccharides from lignocellulosic biomass: A comprehensive review. Carbohydr. Polym..

[6] Arai T., Biely P., Uhliarikova I., Sato N., Makishima S., Mizuno M., Nozaki K., Kaneko S., Amano Y. (2019). Structural characterization of hemicellulose released from corn cob in continuous flow type hydrothermal reactor. J. Biosci. Bioeng..

[7] Isci A., Thieme N., Lamp A., Zverlov V., Kaltschmitt M. (2021). Production of xylo-oligosaccharides from wheat straw using microwave assisted deep eutectic solvent pretreatment. Ind. Crops Prod..

[8] Pattarapisitporn A., Thiangthong N., Inthajak P., Jaichakan P., Panpa W., Klangpetch W. (2021). Production of Xyloligosaccharides from Rice Straw by Microwave-assisted Enzymatic Hydrolysis and Evaluation of Their Prebiotic Properties. Chiang Mai Univ. J. Nat. Sci..

[9] Samanta A.K., Jayapal N., Jayaram C., Roy S., Kolte A.P., Senani S., Sridhar M. (2015). Xylooligosaccharides as prebiotics from agricultural by-products: Production and applications. Bioact. Carbohydr. Diet. Fibre.

[10] Bian J., Peng F., Peng X.P., Peng P., Xu F., Sun R.C. (2013). Structural features and antioxidant activity of xylooligosaccharides enzymatically produced from sugarcane bagasse. Bioresour. Technol..

[11] Amorim C., Silverio S.C., Prather K.L.J., Rodrigues L.R. (2019). From lignocellulosic residues to market: Production and commercial potential of xylooligosaccharides. Biotechnol. Adv..

[12] Rashid R., Sohail M. (2021). Xylanolytic Bacillus species for xylooligosaccharides production: A critical review. Bioresour. Bioprocess..

[13] Shimoda K., Hamada H., Hamada H. (2011). Synthesis of xylooligosaccharides of daidzein and their anti-oxidant and anti-allergic activities. Int. J. Mol. Sci..

[14] Boonchuay P., Wongpoomchai R., Jaturasitha S., Mahatheeranont S., Watanabe M., Chaiyaso T. (2021). Prebiotic properties, antioxidant activity, and acute oral toxicity of xylooligosaccharides derived enzymatically from corncob. Food Biosci..

[15] Chen Y., Xie Y., Ajuwon K.M., Zhong R., Li T., Chen L., Zhang H., Beckers Y., Everaert N. (2021). Xylo-Oligosaccharides, Preparation and Application to Human and Animal Health: A Review. Front. Nutr..

[16] Cardoso B.B., Amorim C., Silvério S.C., Rodrigues L.R. (2021). Novel and emerging prebiotics: Advances and opportunities. Advances in Food and Nutrition Research.

[17] Iliev I., Vasileva T., Bivolarski V., Momchilova A., Ivanova I. (2020). Metabolic Profiling of Xylooligosaccharides by Lactobacilli. Polymers.

[18] Finegold S.M., Li Z., Summanen P.H., Downes J., Thames G., Corbett K., Dowd S., Krak M., Heber D. (2014). Xylooligosaccharide increases bifidobacteria but not lactobacilli in human gut microbiota. Food Funct..

[19] Yang J., Summanen P.H., Henning S.M., Hsu M., Lam H., Huang J., Tseng C.H., Dowd S.E., Finegold S.M., Heber D. (2015). Xylooligosaccharide supplementation alters gut bacteria in both healthy and prediabetic adults: A pilot study. Front. Physiol..

[20] Sheu W.H., Lee I.T., Chen W., Chan Y.C. (2008). Effects of xylooligosaccharides in type 2 diabetes mellitus. J. Nutr. Sci. Vitaminol..

[21] Li F., Li Q., Zhang Y., Zhou X., Yi R., Zhao X. (2021). Effects of Xylooligosaccharides on Lipid Metabolism, Inflammation, and Gut Microbiota in C57BL/6J Mice Fed a High-Fat Diet. Front. Pharmacol..

[22] Chen H.H., Chen Y.K., Chang H.C., Lin S.Y. (2012). Immunomodulatory Effects of Xylooligosaccharides. Food Sci. Technol. Res..

[23] Fei Y., Wang Y., Pang Y., Wang W., Zhu D., Xie M., Lan S., Wang Z. (2019). Xylooligosaccharide Modulates Gut Microbiota and Alleviates Colonic Inflammation Caused by High Fat Diet Induced Obesity. Front. Physiol..

[24] Hansen C.H., Frokiaer H., Christensen A.G., Bergstrom A., Licht T.R., Hansen A.K., Metzdorff S.B. (2013). Dietary xylooligosaccharide downregulates IFN-gamma and the low-grade inflammatory cytokine IL-1beta systemically in mice. J. Nutr..

[25] Hintikka J., Lensu S., Mäkinen E., Karvinen S., Honkanen M., Lindén J., Garrels T., Pekkala S., Lahti L. (2021). Xylo-Oligosaccharides in Prevention of Hepatic Steatosis and Adipose Tissue Inflammation: Associating Taxonomic and Metabolomic Patterns in Fecal Microbiomes with Biclustering. Int. J. Environ. Res. Public Health.

[26] Ando H., Ohba H., Sakaki T., Takamine K., Kamino Y., Moriwaki S., Bakalova R., Uemura Y., Hatate Y. (2004). Hot-compressed-water decomposed products from bamboo manifest a selective cytotoxicity against acute lymphoblastic leukemia cells. Toxicol. Vitr..

[27] Maeda R., Ida T., Ihara H., Sakamoto T. (2014). Induction of Apoptosis in MCF-7 Cells by β-1,3-Xylooligosaccharides Prepared fromCaulerpa lentillifera. Biosci. Biotechnol. Biochem..

[28] Ghosh A., Chandra A., Dhar A., Shukla P., Baishya D. (2021). Multi-efficient thermostable endoxylanase from Bacillus velezensis AG20 and its production of xylooligosaccharides as efficient prebiotics with anticancer activity. Process Biochem..

[29] Hsu C.-K., Liao J.-W., Chung Y.-C., Hsieh C.-P., Chan Y.-C. (2004). Xylooligosaccharides and Fructooligosaccharides Affect the Intestinal Microbiota and Precancerous Colonic Lesion Development in Rats. J. Nutr..

[30] Aachary A.A., Gobinath D., Srinivasan K., Prapulla S.G. (2015). Protective effect of xylooligosaccharides from corncob on 1,2-dimethylhydrazine induced colon cancer in rats. Bioact. Carbohydr. Diet. Fibre.

[31] Ávila P.F., Martins M., de Almeida Costa F.A., Goldbeck R. (2020). Xylooligosaccharides production by commercial enzyme mixture from agricultural wastes and their prebiotic and antioxidant potential. Bioact. Carbohydr. Diet. Fibre.

[32] Synytsya A. (2003). Fourier transform Raman and infrared spectroscopy of pectins. Carbohydr. Polym..

[33] Yuan T.-Q., Xu F., He J., Sun R.-C. (2010). Structural and physico-chemical characterization of hemicelluloses from ultrasound-assisted extractions of partially delignified fast-growing poplar wood through organic solvent and alkaline solutions. Biotechnol. Adv..

[34] Bernardino-Nicanor A., Acosta-García G., Güemes-Vera N., Montañez-Soto J.L., de los Ángeles Vivar-Vera M., González-Cruz L. (2016). Fourier transform infrared and Raman spectroscopic study of the effect of the thermal treatment and extraction methods on the characteristics of ayocote bean starches. J. Food Sci. Technol..

[35] Kacuráková M. (2000). FT-IR study of plant cell wall model compounds: Pectic polysaccharides and hemicelluloses. Carbohydr. Polym..

[36] Fotakis G., Timbrell J.A. (2006). In vitro cytotoxicity assays: Comparison of LDH, neutral red, MTT and protein assay in hepatoma cell lines following exposure to cadmium chloride. Toxicol. Lett..

[37] Sivandzade F., Bhalerao A., Cucullo L. (2019). Analysis of the Mitochondrial Membrane Potential Using the Cationic JC-1 Dye as a Sensitive Fluorescent Probe. Bio-Protocol.

[38] Okamoto M., Hirai H., Taniguchi K., Shimura K., Inaba T., Shimazaki C., Taniwaki M., Imanishi J. (2009). Toll-like Receptors (TLRs) are expressed by myeloid leukaemia cell lines, but fail to trigger differentiation in response to the respective TLR ligands. Br. J. Haematol..

[39] Zhong X., Liu M., Yao W., Du K., He M., Jin X., Jiao L., Ma G., Wei B., Wei M. (2019). Epigallocatechin-3-Gallate Attenuates Microglial Inflammation and Neurotoxicity by Suppressing the Activation of Canonical and Noncanonical Inflammasome via TLR4/NF-κB Pathway. Mol. Nutr. Food Res..

[40] Desideri E., Ciccarone F., Ciriolo M.R. (2019). Targeting Glutathione Metabolism: Partner in Crime in Anticancer Therapy. Nutrients.

[41] Kennedy L., Sandhu J.K., Harper M.-E., Cuperlovic-Culf M. (2020). Role of Glutathione in Cancer: From Mechanisms to Therapies. Biomolecules.

[42] Ferreira de Freitas R., Schapira M. (2017). A systematic analysis of atomic protein–ligand interactions in the PDB. MedChemComm.

[43] Zhang H., Xu Y., Yu S. (2017). Co-production of functional xylooligosaccharides and fermentable sugars from corncob with effective acetic acid prehydrolysis. Bioresour. Technol..

[44] Morais de Carvalho D., Martínez-Abad A., Evtuguin D.V., Colodette J.L., Lindström M.E., Vilaplana F., Sevastyanova O. (2017). Isolation and characterization of acetylated glucuronoarabinoxylan from sugarcane bagasse and straw. Carbohydr. Polym..

[45] Zhang L., Zeng X., Qiu J., Du J., Cao X., Tang X., Sun Y., Li S., Lei T., Liu S. (2019). Spray-dried xylooligosaccharides carried by gum Arabic. Ind. Crops Prod..

[46] Yoshino K., Higashi N., Koga K. (2006). Inhibitory effects of acidic xylooligosaccharide on stress-induced gastric inflammation in mice. Shokuhin Eiseigaku Zasshi. J. Food Hyg. Soc. Jpn..

[47] Gobinath D., Madhu A.N., Prashant G., Srinivasan K., Prapulla S.G. (2010). Beneficial effect of xylo-oligosaccharides and fructo-oligosaccharides in streptozotocin-induced diabetic rats. Br. J. Nutr..

[48] Cochet F., Peri F. (2017). The Role of Carbohydrates in the Lipopolysaccharide (LPS)/Toll-Like Receptor 4 (TLR4) Signalling. Int. J. Mol. Sci..

[49] Fatima N., Akhtar T., Sheikh N. (2017). Prebiotics: A Novel Approach to Treat Hepatocellular Carcinoma. Can. J. Gastroenterol. Hepatol..

[50] Javaid N., Choi S. (2020). Toll-like Receptors from the Perspective of Cancer Treatment. Cancers.

[51] Fernández-Lainez C., Akkerman R., Oerlemans M.M.P., Logtenberg M.J., Schols H.A., Silva-Lagos L.A., López-Velázquez G., de Vos P. (2022). β(2→6)-Type fructans attenuate proinflammatory responses in a structure dependent fashion via Toll-like receptors. Carbohydr. Polym..

[52] Zhang Y., Liang X., Bao X., Xiao W., Chen G. (2022). Toll-like receptor 4 (TLR4) inhibitors: Current research and prospective. Eur. J. Med. Chem..

[53] Hsu R.Y.C., Chan C.H.F., Spicer J.D., Rousseau M.C., Giannias B., Rousseau S., Ferri L.E. (2011). LPS-Induced TLR4 Signaling in Human Colorectal Cancer Cells Increases β1 Integrin-Mediated Cell Adhesion and Liver Metastasis. Cancer Res..

[54] Guillot L., Medjane S., Le-Barillec K., Balloy V., Danel C., Chignard M., Si-Tahar M. (2004). Response of Human Pulmonary Epithelial Cells to Lipopolysaccharide Involves Toll-like Receptor 4 (TLR4)-dependent Signaling Pathways. J. Biol. Chem..

[55] Zhu Z., Du S., Du Y., Ren J., Ying G., Yan Z. (2018). Glutathione reductase mediates drug resistance in glioblastoma cells by regulating redox homeostasis. J. Neurochem..

[56] DuBois M., Gilles K.A., Hamilton J.K., Rebers P.A., Smith F. (2002). Colorimetric Method for Determination of Sugars and Related Substances. Anal. Chem..

[57] Blumenkrantz N., Asboe-Hansen G. (1973). New method for quantitative determination of uronic acids. Anal. Biochem..

[58] Singleton V.L., Rossi J.A.J. (1965). Colorimetry of total phenolics with phosphomolybdic-phosphotungstic acid reagents. Am. J. Enol. Vitic..

[59] McComb E.A., McCready R.M. (2002). Determination of Acetyl in Pectin and in Acetylated Carbohydrate Polymers. Anal. Chem..

[60] Ou B., Hampsch-Woodill M., Prior R.L. (2001). Development and Validation of an Improved Oxygen Radical Absorbance Capacity Assay Using Fluorescein as the Fluorescent Probe. J. Agric. Food Chem..

[61] Teneva D., Pencheva D., Petrova A., Ognyanov M., Georgiev Y., Denev P. (2022). Addition of Medicinal Plants Increases Antioxidant Activity, Color, and Anthocyanin Stability of Black Chokeberry (*Aronia melanocarpa*) Functional Beverages. Plants.

[62] Ou B., Hampsch-Woodill M., Flanagan J., Deemer E.K., Prior R.L., Huang D. (2002). Novel Fluorometric Assay for Hydroxyl Radical Prevention Capacity Using Fluorescein as the Probe. J. Agric. Food Chem..

[63] Edmondson J.M., Armstrong L.S., Martinez A.O. (1988). A rapid and simple MTT-based spectrophotometric assay for determining drug sensitivity in monolayer cultures. J. Tissue Cult. Methods.

[64] Repetto G., del Peso A., Zurita J.L. (2008). Neutral red uptake assay for the estimation of cell viability/cytotoxicity. Nat. Protoc..

[65] Marques S.M., Esteves da Silva J.C.G. (2009). Firefly bioluminescence: A mechanistic approach of luciferase catalyzed reactions. IUBMB Life.

[66] Dallakyan S., Olson A.J. (2015). Small-Molecule Library Screening by Docking with PyRx. Chemical Biology.

[67] Dhanda S.K., Mahajan S., Paul S., Yan Z., Kim H., Jespersen M.C., Jurtz V., Andreatta M., Greenbaum J.A., Marcatili P. (2019). IEDB-AR: Immune epitope database—analysis resource in 2019. Nucleic Acids Res..

[68] Temml V., Kaserer T., Kutil Z., Landa P., Vanek T., Schuster D. (2014). Pharmacophore modeling for COX-1 and -2 inhibitors with LigandScout in comparison to Discovery Studio. Future Med. Chem..

[69] Yang Z., Lasker K., Schneidman-Duhovny D., Webb B., Huang C.C., Pettersen E.F., Goddard T.D., Meng E.C., Sali A., Ferrin T.E. (2012). UCSF Chimera, MODELLER, and IMP: An integrated modeling system. J. Struct. Biol..

[70] O’Boyle N.M., Banck M., James C.A., Morley C., Vandermeersch T., Hutchison G.R. (2011). Open Babel: An open chemical toolbox. J. Cheminform..

